# Efficient preconditioning strategies for accelerating GMRES in block-structured nonlinear systems for image deblurring

**DOI:** 10.1371/journal.pone.0322146

**Published:** 2025-06-25

**Authors:** Rizwan Khalid, Shahbaz Ahmad, Mohamed Medani, Yahia Said, Iftikhar Ali

**Affiliations:** 1 Abdus Salam School of Mathematical Sciences, Government College University, Lahore, Pakistan; 2 Applied College of Muhayil Aseer, King Khalid University, Muhayil Aseer, Saudi Arabia; 3 Center for Scientific Research and Entrepreneurship, Northern Border University, 73213, Arar, Saudi Arabia; 4 Department of Mathematics, University of Hafr Al Batin, Hafr Al Batin, Saudi Arabia; Northwestern Polytechnical University, CHINA

## Abstract

We propose an efficient preconditioning strategy to accelerate the convergence of Krylov subspace methods, specifically for solving complex nonlinear systems with a block five-by-five structure, commonly found in cell-centered finite difference discretizations for image deblurring using mean curvature techniques. Our method introduces two innovative preconditioned matrices, analyzed spectrally to show a favorable eigenvalue distribution that accelerates convergence in the Generalized Minimal Residual (GMRES) method. This technique significantly improves image quality, as measured by peak signal-to-noise ratio (PSNR), and demonstrates faster convergence compared to traditional GMRES, requiring minimal CPU time and few iterations for exceptional deblurring performance. The preconditioned matrices’ eigenvalues cluster around 1, indicating a beneficial spectral distribution. The source code is available at https://github.com/shahbaz1982/Precondition-Matrix.

## 1 Introduction

For the past three decades, the importance of research in image deblurring has focused on nonlinear variational methods. The use of these techniques on large, blurry, and noisy images faces two primary obstacles: a large-scale system of equations and nonlinearity, which arise when discretization is applied after linearization. The purpose of this paper is to explain the challenges associated with these calculations. Mean Curvature (MC) regularization is a nonlinear variational model commonly used in image deblurring. It is used and explained in detail in previous work [17, 34, 40, 42, 43]. MC-based regularization models have the ability to minimize the staircase effect and preserve edges, which is why they are often used in image deblurring during the image recovery process. Discretizing the Euler-Lagrange equations leads to a large, nonlinear system that is often ill-conditioned ,which poses significant challenges for the stability and convergence of computational methods. In the mean curvature-driven system, the Jacobian matrix displays a segmented, banded configuration with considerable bandwidth, highlighting the need for a robust and efficient computational strategy. The next section introduces the 5×5 block structure that arises from the discretization of the mean curvature-based image deblurring problem, as discussed in [20–22].


$$Au=[\begin{array}{ccccc} K_{h}^{*}K_{h} & -\alpha A_{h} & 0 & \alpha B_{h}^{*} & -\alpha B_{h}^{*}\\ 0 & I_{h} & -B_{h}^{*} & 0 & 0\\ B_{h} & 0 & D_{h} & 0 & 0\\ 0 & B_{h} & 0 & D_{h} & 0\\ 0 & 0 & -C_{h} & 0 & D_{h} \end{array}][\begin{array}{c} u_{1}\\ u_{2}\\ u_{3}\\ u_{4}\\ u_{5} \end{array}]=[\begin{array}{c} K_{h}^{*}z\\ 0\\ 0\\ 0\\ 0 \end{array}]=b.$$

Addressing these types of systems presents a significant challenge for computational techniques ,despite using established techniques like Krylov subspace methods, such as the Generalized Minimal Residual (GMRES) method. These methods often exhibit slow convergence, reducing efficiency. A promising solution is the use of preconditioning techniques, as discussed in recent studies [4, 5, 10, 24, 29]. In practice, preconditioners are often applied with some inexactness through inner iterations, frequently using methods such as Flexible GMRES (FGMRES) [13, 26]. Iterative methods rely on specific parameters, but the optimal choice of these parameters may not always be ideal for Krylov subspace methods with preconditioning matrices.

This paper presents novel block preconditioners designed specifically for the five-by-five block structure of the mean curvature-based image sharpening problem , eliminating the necessity of supplementary parameters . These preconditioners aim to improve efficiency while simplifying the solution process. We introduce an innovative partitioning strategy for the coefficient matrix *A* , ensuring stable and consistent convergence of the iterative method without specific conditions. This results in the development of two novel preconditioners that notably improve the convergence rates of Krylov subspace techniques. The preconditioned matrices exhibit a clustering of eigenvalues around 1, suggesting improved convergence for the GMRES method with preconditioning. The primary contributions of this study are summarized below, highlighting the novel approaches and advancements introduced. These contributions enhance the performance and efficiency of the proposed methods.

We introduce novel block preconditioners for the MC-based image deblurring problem’s five-by-five block structure and an unconditionally convergent partitioning strategy. A frequency-domain analysis ensures better eigenvalue distribution and faster convergence. Comprehensive experiments validate the theoretical findings. We also compare our approach with state-of-the-art methods. The organization of the paper is as follows: Sect 2 presents the problem formulation, the discretization technique, and the 5×5 block structure related for image deblurring methods rooted in mean curvature principles restoration issue. Sect 3 provides details on Krylov subspace methods. Various iterative methods and the proposed preconditioners, along with an analysis of the eigenvalue distribution, are discussed in Sect 4. Sect 5 evaluates the performance of the proposed preconditioners in comparison with existing numerical techniques for the image deblurring problem. Sect 6 concludes the study.

## 2 Problem description

The main objective of the present study is to develop a robust solution to the complex and challenging problem of image deblurring, aiming to significantly improve image clarity and quality , focusing on developing effective solutions to improve image quality and clarity , and We begin by providing a brief overview of the problem, highlighting its key aspects and challenges. This paper is dedicated to tackling the challenge of image deblurring, aiming to develop more effective methods for restoring image clarity and details , starting with a brief summary of the problem. From a mathematical perspective, The mathematical formulation relating The connection between the original image *u* and the observed (measured) image *z* is represented as follows:

$$z=\vec{K}u+\text{E}$$ (1)

In this formula, E denotes the noise function, which can appear as different forms of noise, salt-and-pepper noise, including Gaussian noise, and so on. Within the context of this paper, We focus specifically on Gaussian noise. The blurring operator $\vec{K}$ is characterized as a Fredholm integral operator of the first kind, which models the blurring process in image processing through integration over a specific domain. This operator is fundamental in defining the transformation from the original image to the blurred image , which acts to model the degradation process in image restoration:


$$(\vec{K}u)(x)=\int_{\Omega }k(x,y)u(y)\;dx,\;x\in \Omega$$

This operator exhibits a property known as translation invariance, meaning its behavior remains consistent when applied across different spatial locations. The problem formulated in (Eq 1) is regarded as ill-posed [2, 31–33, 35], mainly because the operator $\vec{K}$ exhibits compactness, which leads to challenges in obtaining a stable and well-defined solution. This compactness introduces challenges in the problem’s solvability, leading to instability in the numerical solution process. The lack of a well-defined solution, coupled with the operator’s inherent properties, results in a system where small perturbations in the data can lead to significant deviations in the outcome. These characteristics highlight the need for specialized techniques to stabilize and solve the problem effectively. Let Ω denote a square region within ${\mathbb{R}}^{2}$, where the function *u* defined on Ω represents the intensity values of the image. In this context, *u* serves as a spatially dependent variable that encodes the brightness or color intensity of each pixel in the image. This intensity function *u* is essential for describing the underlying features of the image in a continuous domain, and it is the target of reconstruction or restoration processes in image deblurring tasks. A point in the domain Ω is denoted by 𝐱=(x,y), where $|𝐱|=\sqrt{x^{2}+y^{2}}$ represents the Euclidean distance of the point from the origin, also known as the Euclidean norm. Additionally, ‖·‖ signifies the $L_{2}(\Omega )$ norm, which is a standard measure used to quantify the magnitude of functions or vectors within the square domain Ω. (Eq 1) describes a problem of inversion, where the goal is to reconstruct the true image *u* from the observed image data *z*. This task is classified as ill-posed, as it lacks guarantees of uniqueness or stability in the solution without incorporating further regularization techniques. This arises due to the inherent instability in the reconstruction process, which is often sensitive to small perturbations or noise in the observed data, as discussed in [2, 35].

A frequently adopted strategy to enhance the stability of the image deblurring procedure is the inclusion of a regularization term based on mean curvature (MC) [16, 17, 19, 30, 34, 40, 42, 43]. This regularization technique aims to preserve important features in the image while minimizing unnecessary noise. The MC functional is mathematically formulated as follows:


$$\int_{\Omega }\kappa (u{)}^{2}dx=\int_{\Omega }(\nabla \cdot \frac{\nabla u}{\left|\nabla u\right|}{)}^{2}dx.$$

As a result, the original problem outlined in (1) is reformulated into an optimization task where the goal is to identify the image *u* that minimizes the following functional. This revised formulation incorporates regularization to achieve a balance between fidelity to the observed data and smoothness in the reconstructed image:

$$F(u)=\frac{1}{2}\|\vec{K}u-z{\|}^{2}+\frac{\alpha }{2}\int_{\Omega }\kappa (u{)}^{2}dx.$$ (2)

Within this approach, the regularization constant α>0 plays a pivotal role in balancing the data accuracy and the smoothness of the solution. The stability and solvability of the problem described by (Eq 2) are examined in the context of synthetic image denoising, as outlined in [42]. This examination offers essential understanding of the key factors influencing the method’s performance, highlighting both its advantages and possible drawbacks. The Euler-Lagrange equations corresponding to the problem defined in (Eq 2) are subsequently derived, yielding the essential conditions required for achieving an optimal solution. These conditions are fundamental to understanding the relationship between the variables and guiding the solution process effectively.

$${\vec{K}}^{*}(\vec{K}u-z)+\alpha ▽.[\frac{▽\kappa }{\sqrt{{\left|▽u\right|}^{2}+\beta^{2}}}-\frac{▽\kappa .▽u}{(\sqrt{{\left|▽u\right|}^{2}+\beta^{2}}{)}^{3}}▽u]=0\text{in}\Omega ,$$ (3)

$$\kappa (u)=0\text{,}\;\frac{\partial u}{\partial n}=0\;\text{ in }\partial \Omega .$$ (4)

In this context, ${\vec{K}}^{*}$ represents the adjoint of the operator $\vec{K}$, which plays a pivotal role in the optimization process. Additionally, introducing β>0 helps to avoid any issues with non-differentiability at zero, ensuring that the optimization procedure remains smooth and stable. Since κ(u) is second order differentiable functional, consequently, (Eq 3) describes a nonlinear differential equation of fourth order, which captures the core dynamics of the problem in a mathematical framework.

The expression in (3) simplifies to a first-order system

$${\vec{K}}^{*}(\vec{K}u-z)+\alpha ▽.\vec{p}=\alpha ▽.\vec{t},$$ (5)

$$w=▽.\vec{v},$$ (6)

$$\sqrt{{\left|▽u\right|}^{2}+\beta^{2}}\vec{v}=▽u,$$ (7)

$$\sqrt{{\left|▽u\right|}^{2}+\beta^{2}}\vec{p}=▽w,$$ (8)

$$\sqrt{{\left|▽u\right|}^{2}+\beta^{2}}\vec{t}=(▽w.\vec{v})\vec{v},$$ (9)

by the following substitutions


$$\vec{t}=\frac{(▽w.\vec{v})\vec{v}}{\sqrt{{\left|▽u\right|}^{2}+\beta^{2}}}\text{,}\;\vec{p}=\frac{▽w}{\sqrt{{\left|▽u\right|}^{2}+\beta^{2}}},\;w=▽.\vec{v}$$

and


$$\vec{v}=\frac{▽u}{\sqrt{{\left|▽u\right|}^{2}+\beta^{2}}}.$$

In tackling the image deblurring problem through mean curvature, an efficient method is utilized to improve the restoration process. Here, we present a concise description of the discretization discussed in [20–22]. The domain Ω=(0,1)×(0,1) is partitioned into grid units, each having a size of $\delta_{x}\;\times \;\delta_{y}$. The points $\left(x_{j},y_{k}\right)$ represent the centers of the cells, defined as:


$$x_{j}=\left(j-\frac{1}{2}\right)h,\;j=1,2,3,\ldots ,n_{x},$$

$$y_{k}=\left(k-\frac{1}{2}\right)h,\;k=1,2,3,\ldots ,n_{y}.$$

Here, *n*_*x*_ and *n*_*y*_ denote the number of equispaced partitions in the *x* and *y* directions, respectively. For simplicity, we assume $n_{x}=n_{y}$ and $h=\frac{1}{n_{x}}$. The points $\left(x_{j\pm \frac{1}{2}},y_{k}\right)$ and $\left(x_{j},y_{k\pm \frac{1}{2}}\right)$ denote the midpoints of the cell edges:


$$x_{j\pm \frac{1}{2}}=x_{j}\pm \frac{h}{2},\;j=1,2,3,\ldots ,n_{x},$$

$$y_{k\pm \frac{1}{2}}=y_{k}\pm \frac{h}{2},\;k=1,2,3,\ldots ,n_{x}.$$

For each $j,k=1,2,\ldots ,n_{x}$, we define the following subdomains:


$$\delta_{j,k}=(x_{j-1/2},x_{j+1/2})\times (y_{k-1/2},y_{k+1/2}),$$

$$\delta_{j+1/2,k}=(x_{j},x_{j+1})\times (y_{k-1/2},y_{k+1/2}),$$

$$\delta_{j,k+1/2}=(x_{j-1/2},x_{j+1/2})\times (y_{k},y_{k+1}).$$

In the context of discrete functions, we require values at specific points to represent the function δ(x,y) accurately. Let $\delta_{l,m}$ denote the discrete representation of $\delta (x_{l},y_{m})$, where *l* and *m* can take values such as *j*, $j+\frac{1}{2}$, *k*, or $k+\frac{1}{2}$. Note that *j* and *k* are non-negative integers. This definition allows us to compute the required values at discrete points for accurate representation. Specifically, we define the discrete derivatives as:


$$\begin{array}{l} {\left[d_{x}\delta \right]}_{j+1/2,k}=\frac{\delta_{j+1,k}-\delta_{j,k}}{h},{\left[D_{x}\delta \right]}_{j.k}=\frac{\delta_{j+1/2,k}-\delta_{j-1/2,k}}{h},\\ {\left[d_{y}\delta \right]}_{j+1/2,k}=\frac{\delta_{j+1,k}-\delta_{j,k}}{h},{\left[D_{y}\delta \right]}_{j.k}=\frac{\delta_{j+1/2,k}-\delta_{j-1/2,k}}{h}, \end{array}$$

Using the midpoint quadrature approximation, we approximate the operator $\left(Ku)(x_{i},y_{j}\right)$ as:


$$(Ku)(x_{i},y_{j})≅[K_{h}U{]}_{\left(ij\right)}.$$

This partitioning approach facilitates the numerical analysis of the problem. Through the use of a lexicographical ordering of the unknowns,


$$u_{1}=[{\overset{―}{U}}_{11}\;...\;{\overset{―}{U}}_{n_{x}n_{x}}{]}^{t}\text{,}\;u_{2}=[{\overset{―}{W}}_{11}\;...\;{\overset{―}{W}}_{n_{x}n_{x}}{]}^{t},$$

$$u_{3}=[{\overset{―}{V}}_{11}^{x}\;...\;{\overset{―}{V}}_{n_{x}-1n_{x}-1}^{x}\;\;{\overset{―}{V}}_{11}^{y}\;...\;{\overset{―}{V}}_{n_{x}-1n_{x}-1}^{y}{]}^{t},$$

$$u_{4}=[{\overset{―}{P}}_{11}^{x}\;...\;{\overset{―}{P}}_{n_{x}-1n_{x}-1}^{x}\;\;{\overset{―}{P}}_{11}^{y}\;...\;{\overset{―}{P}}_{n_{x}-1n_{x}-1}^{y}{]}^{t},$$

$$\text{and}\;u_{5}=[{\overset{―}{T}}_{11}^{x}\;...\;{\overset{―}{T}}_{n_{x}-1n_{x}-1}^{x}\;\;{\overset{―}{T}}_{11}^{y}\;...\;{\overset{―}{T}}_{n_{x}-1n_{x}-1}^{y}{]}^{t}.$$

Through the use of the CCFD method to solve (Eqs 5)–(9), We arrive at the following system:

$$K^{*}K_{h}u_{1}-\alpha A_{h}u_{2}+\alpha B_{h}^{*}u_{4}-\alpha B_{h}^{*}u_{5}=K_{h}^{*}z,$$ (10)

$$I_{h}u_{2}-B_{h}^{*}u_{3}=0,$$ (11)

$$B_{h}u_{1}+D_{h}u_{3}=0,$$ (12)

$$B_{h}u_{2}+D_{h}u_{4}=0,$$ (13)

$$-C_{h}u_{3}+D_{h}u_{5}=0.$$ (14)

The integral term is approximated using the midpoint quadrature rule. The matrix representations *K*_*h*_, *A*_*h*_, and *I*_*h*_ each have a size of $n_{x}^{2}\;\times \;n_{x}^{2}$. The matrix *B*_*h*_ has a size of $2n_{x}(n_{x}\;-\;1)\;\times \;n_{x}^{2}$. The matrices *C*_*h*_ and *D*_*h*_ each have a size of $2n_{x}(n_{x}\;-\;1)\;\times \;2n_{x}(n_{x}\;-\;1)$. As a result, We arrive at the following system:


$$Au=[\begin{array}{ccccc} K_{h}^{*}K_{h} & -\alpha A_{h} & 0 & \alpha B_{h}^{*} & -\alpha B_{h}^{*}\\ 0 & I_{h} & -B_{h}^{*} & 0 & 0\\ B_{h} & 0 & D_{h} & 0 & 0\\ 0 & B_{h} & 0 & D_{h} & 0\\ 0 & 0 & -C_{h} & 0 & D_{h} \end{array}][\begin{array}{c} u_{1}\\ u_{2}\\ u_{3}\\ u_{4}\\ u_{5} \end{array}]=[\begin{array}{c} K_{h}^{*}z\\ 0\\ 0\\ 0\\ 0 \end{array}]=b.$$

The matrix *K*_*h*_ has a structure known as Block Toeplitz with Toeplitz blocks (BTTB) , which enhances its computational efficiency in large-scale problems , and the product $K_{h}^{*}K_{h}$ is both symmetric and positive definite (SPD), which ensures its stability in numerical computations. The matrix *A*_*h*_ can be expressed as a diagonal matrix, arranged in the following manner:


$$A_{h}=\frac{2(A_{1}+A_{2})}{\beta h},$$

In this framework, the matrices *A*_1_ and *A*_2_ are of size $n_{x}^{2}\;\times \;n_{x}^{2}$ and are specified as follows:


$$A_{1}=\tilde{I}⊗E_{1}\;\text{and}\;A_{2}=E_{1}⊗\tilde{I},$$

where ⊗ represents the tensor product. The identity matrix $\tilde{I}$ has dimensions $n_{x}\;\times \;n_{x}$. The matrix


$$E_{1}=[\begin{array}{cccccc} 1 & & & & & \\ & 0 & & & & \\ & & \ddots & & & \\ & & & & 0 & \\ & & & & & 1 \end{array}],$$

has dimensions $n_{x}\;\times \;n_{x}$. The matrix *B*_*h*_ exhibits the following structure:


$$B_{h}=\frac{1}{h}[\begin{array}{c} B_{1}\\ B_{2} \end{array}]$$

where both *B*_1_ and *B*_2_ have dimensions $n_{x}(n_{x}-1)\;\times \;n_{x}^{2}$, and


$$B_{1}=E_{2}⊗\tilde{I}\;\text{and}\;B_{2}=\tilde{I}⊗E_{2}.\;E_{2}=[\begin{array}{cccccc} 1 & -1 & & & & \\ & 1 & -1 & & & \\ & & \ddots & \ddots & & \\ & & & \ddots & -1 & \\ & & & & 1 & -1 \end{array}],$$

is a matrix with dimensions $(n_{x}-1)\;\times \;n_{x}$. The matrix


$$C_{h}=[\begin{array}{cc} C^{x} & 0\\ 0 & C^{y} \end{array}],$$

The matrix is structured as a diagonal matrix, with its elements derived from the discretization of the term $\left(\nabla w\cdot \vec{v}\right)$. The matrix *C*^*x*^ has dimensions $(n_{x}-1)\;\times \;n_{x}$, while *C*^*y*^ is of size $n_{x}\;\times \;(n_{x}-1)$. In a similar fashion, the matrix *D*_*h*_ is diagonal, with positive values on its diagonal. The diagonal values of *D*_*h*_ are computed by discretizing the expression $\sqrt{{\left|\nabla u\right|}^{2}+\beta^{2}}$. The matrix *D*_*h*_ is structured as follows:


$$D_{h}=[\begin{array}{cc} D^{x} & 0\\ 0 & D^{y} \end{array}].$$

In recent years, significant advancements have been achieved in the development of efficient iterative techniques for solving large block matrix problems [8, 9, 15, 28, 38, 39, 41]. In particular, the integration of Krylov subspace methods with suitable preconditioning strategies has demonstrated remarkable effectiveness in addressing these complex challenges [14, 25, 27].

## 3 Krylov subspace methods

Krylov subspace methods are a class of iterative algorithms used for solving large, sparse systems of linear equations of the form A𝐱=𝐛, where *A* is a nonsingular matrix. These methods construct approximate solutions in a *Krylov subspace*, defined as:


$${𝒦}_{m}(A,{𝐫}_{0})=\text{span}\{{𝐫}_{0},A{𝐫}_{0},A^{2}{𝐫}_{0},\ldots ,A^{m-1}{𝐫}_{0}\},$$

where ${𝐫}_{0}=𝐛-A{𝐱}_{0}$ is the initial residual. Krylov methods iteratively refine the solution by projecting the problem onto this subspace. The GMRES method is a widely used Krylov subspace method for solving nonsymmetric or non-Hermitian systems. At the *m*-th iteration, GMRES computes the solution ${𝐱}_{m}$ that minimizes the residual $\|𝐛-A{𝐱}_{m}{\|}_{2}$ over the subspace ${𝒦}_{m}(A,{𝐫}_{0})$. This is achieved using the Arnoldi process to construct an orthonormal basis of ${𝒦}_{m}$, and then solving a small least-squares problem at each iteration.

GMRES is particularly effective for nonsymmetric systems, but its performance can degrade for ill-conditioned problems. To address this, preconditioning is often employed [3, 4, 6, 10, 29]. To accelerate convergence, GMRES is often coupled with a preconditioner, which transforms the original system A𝐱=𝐛 into


$$M^{-1}A𝐱=M^{-1}𝐛,$$

where *M* is a preconditioner matrix that approximates *A* but is easier to invert.

Consequently, Krylov subspace methods combined with preconditioning are anticipated to show significantly enhanced convergence behavior. Additionally, in the following section, we introduce two innovative preconditioners aimed at accelerating the rate at which Krylov subspace methods converge when applied to the image deblurring problem can be significantly improved with the use of tailored preconditioners.

## 4 Novel preconditioned matrices

This section begins by providing an overview of fundamental concepts and key principles associated with iterative methods, which are commonly employed in numerical computations. These methods serve as the foundation for efficiently The solution of large-scale systems plays a crucial role in numerous applications, particularly in tasks such as image deblurring and various other computational problems. Consider a matrix $A\in {\mathbb{R}}^{n\times n}$, this can be expressed using an arbitrary factorization of *A* as *A* = *P*−*R*, where *P* represents an invertible matrix and *R* represents the residual component that captures the discrepancy. In this factorization, A basic stationary iterative technique employed to solve the system (10)–(14) can be expressed as follows:

$$y^{\left(l+1\right)}=P^{-1}Ry^{\left(l\right)}+P^{-1}c,\;l=0,1,2,....$$ (15)

In the wider framework of iterative techniques, it is well-known that the iterative scheme (15) will ultimately converge, irrespective of the initial value $y^{\left(0\right)}\in {\mathbb{R}}^{n}$, provided that the spectral radius of the iteration matrix *P*^−1^*R* remains strictly below one, i.e., $\rho (P^{-1}R)<1$.

For the first preconditioner *P*_1_, we explore a block factorization of the matrix *A* related to the system (10)–(14), given as follows:

$$A=P_{1}-R_{1}$$ (16)

$$\left[\begin{array}{ccccc} K_{h}^{*}K_{h} & -\alpha A_{h} & 0 & \alpha B_{h}^{*} & -\alpha B_{h}^{*}\\ 0 & I_{h} & -B_{h}^{*} & 0 & 0\\ B_{h} & 0 & D_{h} & 0 & 0\\ 0 & B_{h} & 0 & D_{h} & 0\\ 0 & 0 & -C_{h} & 0 & D_{h} \end{array}\right]$$

$$=[\begin{array}{ccccc} K_{h}^{*}K_{h} & -\alpha A_{h} & 0 & 0 & 0\\ 0 & I_{h} & 0 & 0 & 0\\ B_{h} & 0 & D_{h}+\alpha B(K^{*}K{)}^{-1}AB^{*} & -\alpha B(K^{*}K{)}^{-1}B^{*} & \alpha B(K^{*}K{)}^{-1}B^{*}\\ 0 & B_{h} & B_{h}B_{h}^{*} & D_{h} & 0\\ 0 & 0 & -C_{h} & 0 & D_{h} \end{array}]$$

$$-[\begin{array}{ccccc} 0 & 0 & 0 & -\alpha B_{h}^{*} & \alpha B_{h}^{*}\\ 0 & 0 & B_{h}^{*} & 0 & 0\\ 0 & 0 & \alpha B_{h}(K_{h}^{*}K_{h}{)}^{-1}A_{h}B_{h}^{*} & -\alpha B_{h}(K_{h}^{*}K_{h}{)}^{-1}B_{h}^{*} & \alpha B_{h}(K_{h}^{*}K_{h}{)}^{-1}B_{h}^{*}\\ 0 & 0 & B_{h}B_{h}^{*} & 0 & 0\\ 0 & 0 & 0 & 0 & 0 \end{array}].$$

For the initial preconditioner *P*_1_, we investigate a block decomposition of the coefficient matrix *A* associated with the system (10)–(14), expressed as follows:

$$P_{1}=\left[\begin{array}{cc} Q & \;\;0\\ N & L-NQ^{-1}M \end{array}\right],\;R_{1}=\left[\begin{array}{cc} 0 & \;\;-M\\ 0 & -NQ^{-1}M \end{array}\right]$$ (17)

where


$$Q=\left[\begin{array}{cc} K_{h}^{*}K_{h} & -\alpha A_{h}\\ 0\; & I_{h} \end{array}\right],M=\left[\begin{array}{cc} 0 & \alpha B_{h}^{*}\;-\alpha B_{h}^{*}\\ -B_{h}^{*} & \;0\;\;\;0 \end{array}\right],$$

$$N=\left[\begin{array}{cc} B_{h} & 0\\ 0 & B_{h}\\ 0\; & 0 \end{array}\right],L=\left[\begin{array}{cc} D_{h} & 0\;\;\;0\\ 0 & D_{h}\;\;0\\ -C_{h} & 0\;\;D_{h} \end{array}\right].$$

As a result, the system (10)–(14) naturally leads to the following 2×2 block structure:

$$\underset{\text{A}}{\underbrace{\left[\begin{array}{cc} Q & \;M\\ N & L \end{array}\right]}}\underset{\text{x}}{\underbrace{\left[\begin{array}{c} U_{1}\\ U_{2} \end{array}\right]}}=\underset{\text{b}}{\underbrace{\left[\begin{array}{c} Z_{1}\\ \;Z_{2} \end{array}\right]}},$$ (18)

where $U_{1}=[u_{1}\;u_{2}{]}^{T},U_{1}=[u_{3}\;u_{4}\;u_{5}{]}^{T},Z_{1}=[K_{h}^{*}z\;0{]}^{T}$ and $Z_{2}=[0\;0\;0{]}^{T}$. To solve the system (18) starting with an arbitrary initial guess $y^{0}\in {\mathbb{R}}^{2n(4n-3)}$, We can subsequently formulate the following iterative method

$$y^{\left(l+1\right)}=P_{1}^{-1}R_{1}y^{\left(l\right)}+P_{1}^{-1}b,l=0,1,2,....$$ (19)

The matrix *P*_1_ in (17) can be expressed in its factored form as:

$$P_{1}=\left[\begin{array}{cc} I & \;\;0\\ NQ^{-1} & I \end{array}\right]\left[\begin{array}{cc} Q & \;\;0\\ 0 & L-NQ^{-1}M \end{array}\right].$$ (20)

Given the decomposition described earlier, it is clear that the matrix *P*_1_ is invertible, leading to the following result:

$$P_{1}^{-1}={\left[\begin{array}{cc} Q & \;\;0\\ 0 & L-NQ^{-1}M \end{array}\right]}^{-1}[\begin{array}{cc} I & 0\\ -NQ^{-1} & I \end{array}]$$ (21)

In order to guarantee the convergence of the iterative process (19), we now introduce the following theorem.

**Theorem 4.1**

Consider a symmetric positive definite (SPD) matrix $Q\in {\mathbb{R}}^{2n^{2}\times 2n^{2}}$ and a full-rank matrix $M\in {\mathbb{R}}^{2n^{2}\;\times \;6n(n-1)}$. Then, for any initial guess and α>0, the iterative process (19) converges to the unique solution of (18).

**Proof**

Since $K_{h}^{*}K_{h}$ is symmetric positive definite (SPD) and *I* is the identity matrix, it follows that *Q* is SPD. Furthermore, since *B*_*h*_ is of full rank, *M* must also be a full rank matrix. Consequently, from (Eq 21), we have


$$P_{1}^{-1}R_{1}={\left[\begin{array}{cc} Q & \;\;0\\ 0 & L-NQ^{-1}M \end{array}\right]}^{-1}[\begin{array}{cc} I & 0\\ -NQ^{-1} & I \end{array}][\begin{array}{cc} 0 & -M\\ 0 & -NQ^{-1}M \end{array}]$$

$$={\left[\begin{array}{cc} Q & \;\;0\\ 0 & L-NQ^{-1}M \end{array}\right]}^{-1}[\begin{array}{cc} 0 & -M\\ 0 & 0 \end{array}]$$

$$=[\begin{array}{cc} 0 & -Q^{-1}M\\ 0 & 0 \end{array}].$$

That is


$$P_{1}^{-1}R_{1}=[\begin{array}{ccccc} 0 & 0 & \alpha (K_{h}^{*}K_{h}{)}^{-1}A_{h}B_{h}^{*} & -\alpha (K_{h}^{*}K_{h}{)}^{-1}B_{h}^{*} & \alpha (K_{h}^{*}K_{h}{)}^{-1}B_{h}^{*}\\ 0 & 0 & B_{h}^{*} & 0 & 0\\ 0 & 0 & 0 & 0 & 0\\ 0 & 0 & 0 & 0 & 0\\ 0 & 0 & 0 & 0 & 0 \end{array}].$$

Thus, we can conclude that the eigenvalues of the matrix $P_{1}^{-1}R_{1}$ are indeed equal to zero.

Therefore, it follows that $\rho (P_{1}^{-1}R_{1})<1$.

This concludes the proof.

In practical applications, The rate of convergence for the stationary iterative method (19) may not be sufficiently rapid to efficiently solve the system (18). To address this limitation, more advanced techniques may be required to achieve faster convergence. The primary goal is to employ the matrix *P*_1_ as a preconditioner to improve the efficiency of Krylov subspace techniques, thus speeding up the convergence. This method is designed to enhance the overall computational effectiveness of solving the system, including methods like GMRES. To implement the preconditioner *P*_1_, it is necessary to solve a system of equations in the following manner, ensuring that the process remains both effective and efficient in accelerating convergence.

$$P_{1}z=P_{1}[\begin{array}{c} z_{1}\\ z_{2}\\ z_{3}\\ z_{4}\\ z_{5} \end{array}]=[\begin{array}{c} w_{1}\\ w_{2}\\ w_{3}\\ w_{4}\\ w_{5} \end{array}]=w$$ (22)

The simple procedure for calculating $z=P_{1}^{-1}w$ is outlined in Algorithm 1.

**Algorithm 1. Computation of** $z=P_{1}^{-1}w$.

1: Solve $(K_{h}^{*}K_{h})z_{1}=w_{1}+\alpha A_{h}w_{2}$ for *z*_1_;

2: Solve $z_{2}=w_{2}$ for *z*_2_;

3: Solve $(D_{h}+\alpha HG)z_{3}=-H(w_{1}+\alpha w_{2}+\alpha B_{h}^{*}D_{h}^{-1}(B_{h}w_{2}-w_{4}+w_{5}))+w_{3}$ for *z*_3_;

4: Solve $D_{h}z_{4}=w_{4}-B_{h}w_{2}-B_{h}B_{h}^{*}z_{3}$ for *z*_4_;

5: Solve $D_{h}z_{5}=w_{5}+C_{h}z_{3}$ for *z*_5_;

     where $H=B_{h}(K_{h}^{*}K_{h}{)}^{-1}$ and $G=A_{h}B_{h}^{*}+B_{h}^{*}D_{h}^{-1}(B_{h}^{*}B_{h}+C_{h})$.

When utilizing the preconditioner *P*_1_, there is a step that requires inverting the matrix $K_{h}^{*}K_{h}$, which can be managed with ease. The matrix $K_{h}^{*}K_{h}$ is symmetric positive definite. Although, $K_{h}^{*}K_{h}$ is full but the blurring operator *K* has translation invariant property, which allows the use of Fast Fourier transformation (FFT) to evaluate $K_{h}^{*}K_{h}u$ in *O*(*nlogn*) operations [36]. Furthermore, when applying the preconditioner *P*_1_, we also encounter a step that involves performing operations with $D_{h}+\alpha HG$, which requires several matrix-vector multiplications. Addressing the linear system with the coefficient matrix $D_{h}+\alpha HG$ becomes significantly computationally intensive when utilizing the preconditioner *P*_1_, primarily because of the increased complexity associated with the operations required. Nevertheless, given that *D*_*h*_ is a matrix with a diagonal structure where all diagonal elements are strictly positive, it inherently exhibits the characteristic of being symmetric positive definite. This property allows for the efficient computation of exact solutions to the system $D_{h}+\alpha HG$, utilizing methods such as the preconditioned conjugate gradient (PCG) approach for inexact solutions or LU decomposition, thus simplifying the overall system [9, 11, 12, 23]. The inversion of *D*_*h*_ when using *P*_1_ is straightforward since *D*_*h*_ is a diagonal matrix.

We now analyze the eigenvalues of the matrix after applying the preconditioner, $P_{1}^{-1}A$, which offers crucial insights into optimizing the performance and efficiency of Krylov subspace methods.

**Theorem 4.2**

Examine a symmetric positive definite (SPD) matrix $Q\in {\mathbb{R}}^{2n^{2}\times 2n^{2}}$, and let $M\in {\mathbb{R}}^{2n^{2}\times 6n(n-1)}$ represent a matrix of full rank. Then, for the matrix *A*, the preconditioner *P*_1_ fulfills the subsequent conditions:


$$\sigma \left(P_{1}^{-1}A\right)=\{1\}.$$

In this case, The symbol σ(·) represents the collection of eigenvalues associated with a matrix.

**Proof**

It can be readily confirmed that


$$P_{1}^{-1}A=I-P_{1}^{-1}R_{1}=[\begin{array}{ccccc} I & 0 & -\alpha (K_{h}^{*}K_{h}{)}^{-1}A_{h}B_{h}^{*} & \alpha (K_{h}^{*}K_{h}{)}^{-1}B_{h}^{*} & -\alpha (K_{h}^{*}K_{h}{)}^{-1}B_{h}^{*}\\ 0 & I & -B_{h}^{*} & 0 & 0\\ 0 & 0 & I & 0 & 0\\ 0 & 0 & 0 & I & 0\\ 0 & 0 & 0 & 0 & I \end{array}].$$

This concludes the proof.

Building upon the previous eigenvalue analysis, it becomes evident that the preconditioned matrix has a minimal polynomial of degree five. This implies that utilizing a technique like GMRES with this preconditioner will lead to exact convergence within a maximum of five iterations.

To further enhance the convergence behavior of the GMRES method, we introduce a second preconditioner, labeled *P*_2_. In order to implement this, we examine a block decomposition of the matrix *A* associated with the system (10)–(14) , which can be represented as:

$$A=P_{2}-R_{2}$$ (23)

$$\left[\begin{array}{ccccc} K_{h}^{*}K_{h} & -\alpha A_{h} & 0 & \alpha B_{h}^{*} & -\alpha B_{h}^{*}\\ 0 & I_{h} & -B_{h}^{*} & 0 & 0\\ B_{h} & 0 & D_{h} & 0 & 0\\ 0 & B_{h} & 0 & D_{h} & 0\\ 0 & 0 & -C_{h} & 0 & D_{h} \end{array}\right]$$

$$=[\begin{array}{ccccc} K_{h}^{*}K_{h} & -\alpha A_{h} & 0 & 2\alpha B_{h}^{*} & -2\alpha B_{h}^{*}\\ 0 & I_{h} & -2B_{h}^{*} & 0 & 0\\ B_{h} & 0 & D_{h}-\alpha B(K^{*}K{)}^{-1}AB^{*} & \alpha B(K^{*}K{)}^{-1}B^{*} & -\alpha B(K^{*}K{)}^{-1}B^{*}\\ 0 & B_{h} & -BB^{*} & D_{h} & 0\\ 0 & 0 & -C_{h} & 0 & D_{h} \end{array}]$$

$$-[\begin{array}{ccccc} 0 & 0 & 0 & \alpha B_{h}^{*} & -\alpha B_{h}^{*}\\ 0 & 0 & -B_{h}^{*} & 0 & 0\\ 0 & 0 & -\alpha B_{h}(K_{h}^{*}K_{h}{)}^{-1}A_{h}B_{h}^{*} & \alpha B_{h}(K_{h}^{*}K_{h}{)}^{-1}B_{h}^{*} & -\alpha B_{h}(K_{h}^{*}K_{h}{)}^{-1}B_{h}^{*}\\ 0 & 0 & -B_{h}B_{h}^{*} & 0 & 0\\ 0 & 0 & 0 & 0 & 0 \end{array}].$$

By applying the substitution outlined above, we can express *P*_2_ and *R*_2_ as follows:

$$P_{2}=\left[\begin{array}{cc} Q & \;\;2M\\ N & L+NQ^{-1}M \end{array}\right],\;R_{2}=\left[\begin{array}{cc} 0 & \;\;M\\ 0 & NQ^{-1}M \end{array}\right]$$ (24)

In order to solve the system (18) starting from an arbitrary initial estimate $y^{0}\in {\mathbb{R}}^{2n(4n-3)}$, the following iterative approach can be applied:

$$y^{\left(l+1\right)}=P_{2}^{-1}R_{2}y^{\left(l\right)}+P_{2}^{-1}b,l=0,1,2,....$$ (25)

The matrix *P*_2_ in (24) can be written as:

$$P_{2}=\left[\begin{array}{cc} I & \;\;0\\ NQ^{-1} & I \end{array}\right]\left[\begin{array}{cc} Q & \;\;0\\ 0 & L-NQ^{-1}M \end{array}\right]\left[\begin{array}{cc} I & \;\;2Q^{-1}M\\ 0 & I \end{array}\right].$$ (26)

The above decomposition yields the following result:

$$P_{2}^{-1}=[\begin{array}{cc} I & -2Q^{-1}M\\ 0 & I \end{array}]{\left[\begin{array}{cc} Q & \;\;0\\ 0 & L-NQ^{-1}M \end{array}\right]}^{-1}[\begin{array}{cc} I & 0\\ -NQ^{-1} & I \end{array}]$$ (27)

We present the following theorem, which provides the guarantee for the convergence of the iterative technique (25).

**Theorem 4.3**

Consider a symmetric positive definite (SPD) matrix $Q\in {\mathbb{R}}^{2n^{2}\times 2n^{2}}$, and let $M\in {\mathbb{R}}^{2n^{2}\times 6n(n-1)}$ be a matrix of full rank. Under these conditions, the iterative process described by (Eq 25), For any arbitrary initial guess and α>0, the method is assured to converge to the unique solution of the system (18).

**Proof**

Since $K_{h}^{*}K_{h}$ is symmetric positive definite (SPD) and *I* is the identity matrix, it follows that *Q* must also be SPD. Furthermore, as *B*_*h*_ is a full-rank matrix, we can conclude that *M* is also a matrix of full rank. Therefore, based on (Eq 27), we can deduce the following:


$$P_{2}^{-1}R_{2}=[\begin{array}{cc} I & -2Q^{-1}M\\ 0 & I \end{array}]{\left[\begin{array}{cc} Q & \;\;0\\ 0 & L-NQ^{-1}M \end{array}\right]}^{-1}[\begin{array}{cc} I & 0\\ -NQ^{-1} & I \end{array}][\begin{array}{cc} 0 & M\\ 0 & NQ^{-1}M \end{array}]$$

$$=[\begin{array}{cc} I & -2Q^{-1}M\\ 0 & I \end{array}]{\left[\begin{array}{cc} Q & \;\;0\\ 0 & L-NQ^{-1}M \end{array}\right]}^{-1}[\begin{array}{cc} 0 & M\\ 0 & 0 \end{array}]$$

$$=[\begin{array}{cc} I & -2Q^{-1}M\\ 0 & I \end{array}][\begin{array}{cc} 0 & Q^{-1}M\\ 0 & 0 \end{array}]$$

$$=[\begin{array}{cc} 0 & Q^{-1}M\\ 0 & 0 \end{array}].$$

That is


$$P_{2}^{-1}R_{2}=[\begin{array}{ccccc} 0 & 0 & -\alpha (K_{h}^{*}K_{h}{)}^{-1}A_{h}B_{h}^{*} & \alpha (K_{h}^{*}K_{h}{)}^{-1}B_{h}^{*} & -\alpha (K_{h}^{*}K_{h}{)}^{-1}B_{h}^{*}\\ 0 & 0 & -B_{h}^{*} & 0 & 0\\ 0 & 0 & 0 & 0 & 0\\ 0 & 0 & 0 & 0 & 0\\ 0 & 0 & 0 & 0 & 0 \end{array}].$$

We have demonstrated that the eigenvalues of the matrix $P_{2}^{-1}R_{2}$ are equal to zero.

As a result, we have $\rho (P_{2}^{-1}R_{2})<1$.

Thus, the proof is now complete.

We now present the following theorem regarding the eigenvalues of the preconditioned matrix $P_{2}^{-1}A$.

**Theorem 4.4**

Let $Q\in {\mathbb{R}}^{2n^{2}\times 2n^{2}}$ be a symmetric positive definite (SPD) matrix, and let $M\in {\mathbb{R}}^{2n^{2}\times 6n(n-1)}$ be a full-rank matrix. Then, for the matrix *A*, the preconditioner *P*_2_ satisfies


$$\sigma \left(P_{2}^{-1}A\right)=\{1\}.$$

**Proof**

It is straightforward to confirm that


$$P_{2}^{-1}A=I-P_{2}^{-1}R=[\begin{array}{ccccc} I & 0 & -\alpha (K_{h}^{*}K_{h}{)}^{-1}A_{h}B_{h}^{*} & \alpha (K_{h}^{*}K_{h}{)}^{-1}B_{h}^{*} & -\alpha (K_{h}^{*}K_{h}{)}^{-1}B_{h}^{*}\\ 0 & I & -B_{h}^{*} & 0 & 0\\ 0 & 0 & I & 0 & 0\\ 0 & 0 & 0 & I & 0\\ 0 & 0 & 0 & 0 & I \end{array}].$$

Hence


$$\sigma \left(P_{2}^{-1}A\right)=\{1\}.$$

It is clear that the matrix *P*_2_ exhibits a minimal polynomial of order five. As a result, iterative methods like GMRES, when combined with *P*_2_, are guaranteed to converge to the exact solution in no more than five iterations. To apply the preconditioner *P*_2_, it is necessary to solve a system of linear equations as outlined below.

$$P_{2}z=P_{2}[\begin{array}{c} z_{1}\\ z_{2}\\ z_{3}\\ z_{4}\\ z_{5} \end{array}]=[\begin{array}{c} w_{1}\\ w_{2}\\ w_{3}\\ w_{4}\\ w_{5} \end{array}]=w$$ (28)

The procedure for calculating $z=P_{2}^{-1}w$ is outlined in Algorithm 2.

Algorithm 2 closely resembles Algorithm 1 in terms of its simplicity and structure. Similar to the previous algorithm, this method includes a step that involves operations with the matrices $K_{h}^{*}K_{h}$, *D*_*h*_ and $D_{h}\;+\;\alpha HG$, which can be performed in the same manner as described in Algorithm 1 above.

**Algorithm 2. Procedure for computing** $z=P_{2}^{-1}w$.

1: Solve $(K_{h}^{*}K_{h})z_{1}=w_{1}+\alpha A_{h}w_{2}+2\alpha (A_{h}z_{3}-z_{4}+z_{5})B_{h}^{*}$ for *z*_1_;

2: Solve $z_{2}=w_{2}+2B_{h}^{*}z_{3}$ for *z*_2_;

3: Solve $(D_{h}+\alpha HG)z_{3}=-H(w_{1}+\alpha w_{2}+\alpha B_{h}^{*}D_{h}^{-1}(B_{h}w_{2}-w_{4}+w_{5}))+w_{3}$ for *z*_3_;

4: Solve $D_{h}z_{4}=w_{4}-B_{h}w_{2}-B_{h}B_{h}^{*}z_{3}$ for *z*_4_;

5: Solve $D_{h}z_{5}=w_{5}+C_{h}z_{3}$ for *z*_5_;

     where $H=B_{h}(K_{h}^{*}K_{h}{)}^{-1}$ and $G=A_{h}B_{h}^{*}+B_{h}^{*}D_{h}^{-1}(B_{h}^{*}B_{h}+C_{h})$.

## 5 Numerical experiments

In this section, we conduct a numerical experiment designed to tackle the challenge of image deblurring. Specifically, we evaluate the performance of our proposed *P*_1_-GMRES and *P*_2_-GMRES methods in comparison with some existing image deblurring approaches [3, 7, 18]. This comparison aims to provide valuable insights into the performance and effectiveness of the proposed method in the context of image reconstruction. By evaluating the different preconditioned GMRES techniques, we will assess how well each method contributes to enhancing the quality and accuracy of the reconstructed images. The suggested methodology centers on leveraging the preconditioned Generalized Minimal Residual Method (PGMRES) as a foundational tool. This approach aims to enhance computational efficiency and convergence speed by carefully incorporating the recommended preconditioner throughout the iterative process. This method is instrumental in enhancing both the efficiency and convergence rate of the iterative process. By utilizing this preconditioned approach, the computational load is effectively managed, leading to faster and more reliable convergence. To address the inherent nonlinearity in the MC model, the Fixed Point Iteration (FPI) method is first applied to the system (10)–(14) for numerical evaluation. This step is crucial for stabilizing the solution procedure and establishing a basis for more precise iterative improvements. Subsequently, the PGMRES algorithm is applied to enhance convergence speed and boost computational efficiency. The numerical experiments in this study were performed on a system with the following specifications: The system used for the numerical experiments is equipped with an Intel^®^ Core^TM^ i5-6300U processor running at 2.40 GHz, 8.00 GB of RAM, and MATLAB R2022a. This configuration was chosen to maintain consistent performance throughout the trials and ensure reliable benchmarking of the iterative methods. The Peak Signal-to-Noise Ratio (PSNR) is a fundamental measure for evaluating the quality of the restored images. It determines how closely the reconstructed image corresponds to the original by comparing their differences. A higher PSNR indicates a greater similarity between the two images, implying a higher restoration quality and better preservation of the original details.

**Example 1**

**Fig 1:**
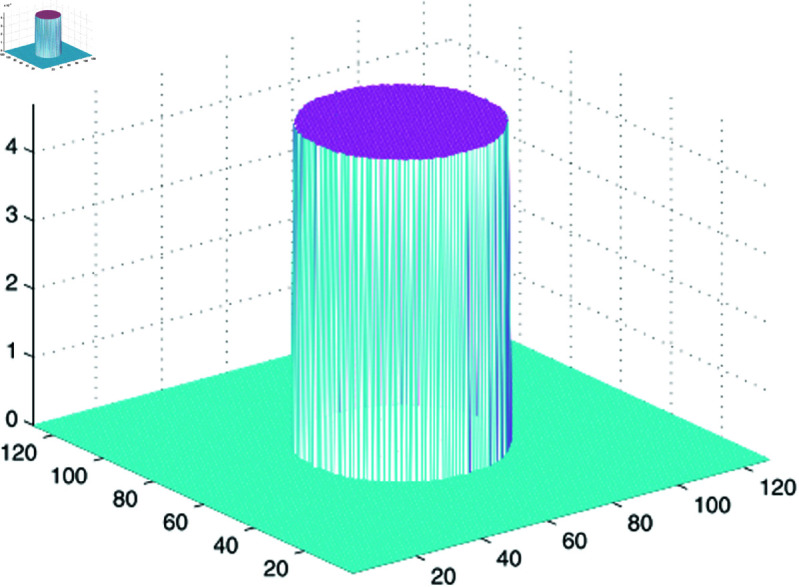
Kernel *ke*_−_*gen*(120,40,4).

In this work, the Clown image , Cameraman image , Boats image and Moon image serves as a standard example, with various feature visualizations displayed in Figs 5, 6, 7, and 8, respectively. Each subfigure in Figs 5, 6, 7, and 8 has dimensions of 512×512. Here, we compare the performance of several methods, including GMRES, *P*_1_-GMRES, *P*_2_-GMRES, and *P*_*S*_-GMRES, as outlined in [3]. A stopping criterion with a tolerance of $\text{tol}=1\;\times \;10^{-7}$ was included in the proposed numerical approach. For the numerical simulations, the *ke*_−_*gen*(*N*,300,5) kernel was utilized, with parameters $\alpha =1\;\times \;10^{-8}$ and β=0.1, as outlined in [20–22] . The result of the *ke*_−_*gen*(120,40,4) kernel is shown in Fig 1. It represents a circular Gaussian kernel with a size of 120×120, a radius of *r* = 40, and a standard deviation of σ=4.

**Fig 2:**
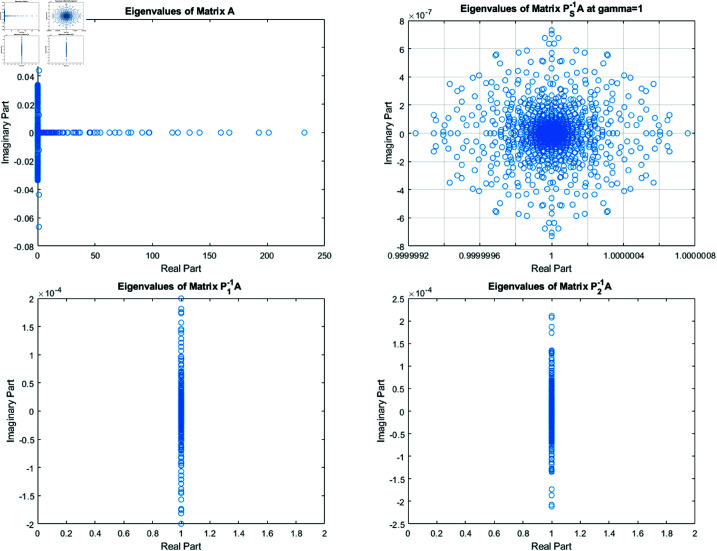
Distribution of eigenvalues for the matrices A, $P_{s}^{-1}A$, $P_{1}^{-1}A$, and $P_{2}^{-1}A$ for Clown image of size 512×512.

**Fig 3:**
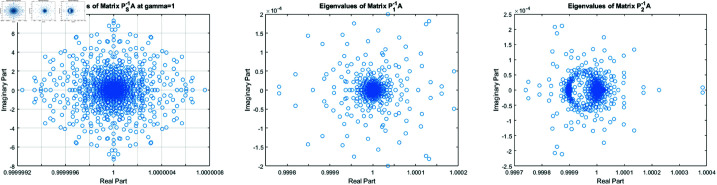
Zoom out figures of eigenvalues distribution of matrices $P_{s}^{-1}A$ , $P_{1}A^{-1}$ and $P_{2}A^{-1}$ for the Clown image of size 512×512.

**Fig 4:**
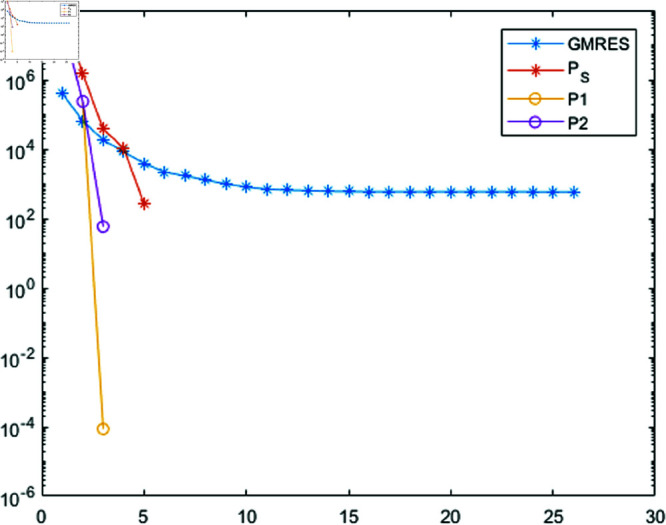
The norm of residuals and the first 25 iterations for GMRES, $P_{S}\text{GMRES}$, $P_{1}\text{GMRES}$, and $P_{2}\text{GMRES}$ are provided for the Clown image of size 512×512.

For the Clown, Cameraman, Boats, and Moon images at resolutions of 128×128, 256×256, and 512×512, the blurred PSNR values, the corresponding deblurred PSNR values and additional details are presented in Tables 1, 2, 3, and 4. These values serve as an objective assessment of the level of degradation observed in the images at each resolution.

**Table 1:** The numerical results for Clown image.

$n_{x}$	Blurred PSNR	Method	Deblurred PSNR	Error	Iterations	CPU-Time
		GMRES	43.1862	9.68429e-5	42	46.3082
		$P_{S}\text{GMRES}$	43.7696	9.95176e-8	2(2)	18.1246
128	18.3566	*P*_1_GMRES	43.7355	2.34672e-9	3(2)	12.3597
		*P*_2_GMRES	43.8219	1.32985e-9	4(3)	10.2062
		GMRES	44.2035	2.40034e-4	57	91.9512
		$P_{S}\text{GMRES}$	43.8356	8.78523e-8	2(4)	27.2659
256	18.2999	*P*_1_GMRES	44.2524	8.25645e-8	3(3)	24.4882
		*P*_2_GMRES	44.3657	6.56914e-8	5(3)	24.6937
		GMRES	42.1252	0.0014332	83	105.6547
		$P_{S}\text{GMRES}$	44.7696	6.95134e-8	7(6)	33.6051
512	18.3764	*P*_1_GMRES	43.0209	7.53245e-8	5(6)	30.6784
		*P*_2_GMRES	43.0039	2.25847e-7	5(3)	29.2521

**Table 2:** The numerical results for Cameraman image.

$n_{x}$	Blurred PSNR	Method	Deblurred PSNR	Error	Iterations	CPU-Time
		GMRES	42.3491	9.67123e-5	41	46.3198
		*P*_*S*_GMRES	42.9965	9.01145e-8	2(3)	16.1567
128	18.6322	*P*_1_GMRES	42.6654	2.12567e-9	3(3)	11.1779
		*P*_2_GMRES	42.2678	1.19876e-9	3(6)	11.2178
		GMRES	43.2678	2.98763e-4	54	91.2678
		*P*_*S*_GMRES	42.1945	8.91287e-8	2(4)	26.7762
256	17.8172	*P*_1_GMRES	43.3164	8.49657e-8	3(6)	23.4167
		*P*_2_GMRES	43.2198	6.31343e-8	5(2)	24.1879
		GMRES	41.1353	0.0019662	82	103.7866
		*P*_*S*_GMRES	43.8976	6.99867e-8	7(5)	32.3576
512	18.2650	*P*_1_GMRES	42.0208	7.25678e-7	5(5)	31.9876
		*P*_2_GMRES	42.1239	2.92547e-7	5(2)	28.7147

**Table 3:** The numerical results for Boats image.

$n_{x}$	Blurred PSNR	Method	Deblurred PSNR	Error	Iterations	CPU-Time
		GMRES	44.1978	9.56789e-5	43	46.4089
		*P*_*S*_GMRES	44.8866	9.89765e-8	3(3)	19.1396
128	17.3561	*P*_1_GMRES	44.6387	2.50713e-9	4(3)	11.7896
		*P*_2_GMRES	43.2167	1.89765e-9	4(4)	10.1844
		GMRES	44.1931	2.21347e-4	55	94.9712
		*P*_*S*_GMRES	44.8596	8.67895e-8	4(4)	26.2567
256	17.0984	*P*_1_GMRES	45.3525	8.35655e-8	3(5)	23.9876
		*P*_2_GMRES	45.4657	6.52567e-8	5(4)	25.9809
		GMRES	41.1342	0.0019876	82	107.65789
		*P*_*S*_GMRES	44.5674	6.98523e-8	7(4)	33.2651
512	17.1227	*P*_1_GMRES	43.0209	8.53245e-8	5(6)	31.1234
		*P*_2_GMRES	44.0049	2.55947e-7	5(3)	25.2541

**Table 4:** The numerical results for Moon image.

$n_{x}$	Blurred PSNR	Method	Deblurred PSNR	Error	Iterations	CPU-Time
		GMRES	57.3228	1.26592e-5	51	49.3698
		*P*_*S*_GMRES	57.6523	6.35618e-9	1(3)	22.3271
128	26.1840	*P*_1_GMRES	57.0289	8.39654e-9	4(3)	15.3257
		*P*_2_GMRES	57.3534	7.36577e-9	4(5)	13.0454
		GMRES	55.4063	2.35622e-5	63	97.6264
		*P*_*S*_GMRES	56.3234	9.35794e-9	5(4)	30.7533
256	26.4905	*P*_1_GMRES	55.9958	8.36969e-9	5(7)	25.9912
		*P*_2_GMRES	56.3890	6.60741e-8	5(4)	26.5916
		GMRES	54.2221	0.0035625	91	111.0025
		*P*_*S*_GMRES	54.3495	3.62459e-8	7(9)	39.2864
512	26.6641	*P*_1_GMRES	54.2099	3.21269e-8	5(8)	30.9431
		*P*_2_GMRES	54.9648	9.35287e-7	5(5)	29.1478


**Remarks**

**Fig 5:**
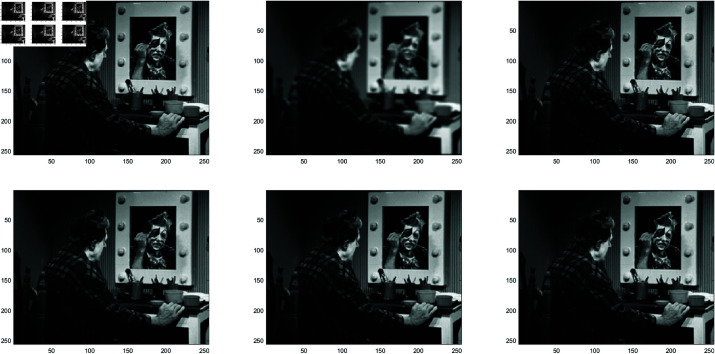
Clown image: (top left to right) original image, blurred image, and deblurred image using GMRES, (bottom left to right) deblurred image using *P*_*s*_*GMRES*, deblurred image using *P*_1_GMRES, and deblurred image using *P*_2_GMRES.

**Fig 6:**
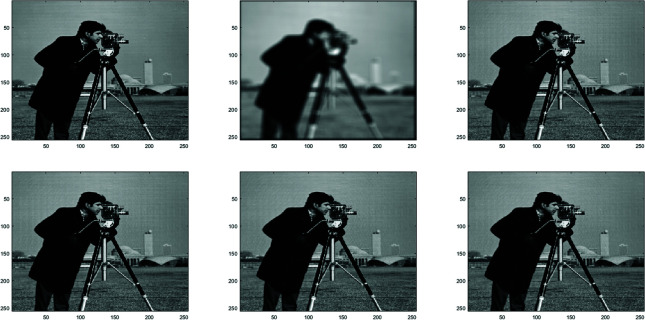
Cameraman image: (top left to right) original image, blurred image, and deblurred image using GMRES, (bottom left to right) deblurred image using *P*_*s*_*GMRES*, deblurred image using *P*_1_GMRES, and deblurred image using *P*_2_GMRES.

**Fig 7:**
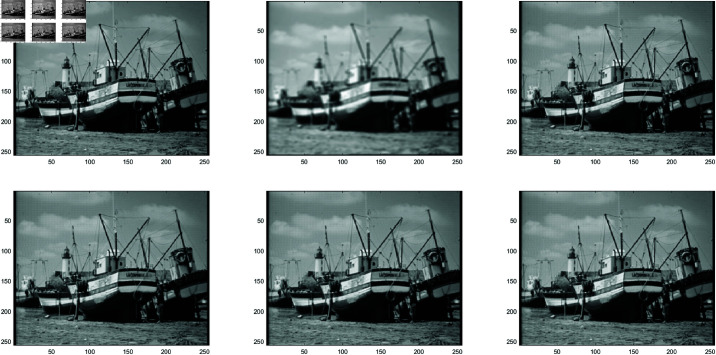
Boats image (Reprinted from [1] under a CC by license, with permission from D.A. Pados, orginal copy right 2008): (top left to right) original image, blurred image, and deblurred image using GMRES, (bottom left to right) deblurred image using *P*_*s*_*GMRES*, deblurred image using *P*_1_GMRES, and deblurred image using *P*_2_GMRES.

**Fig 8:**
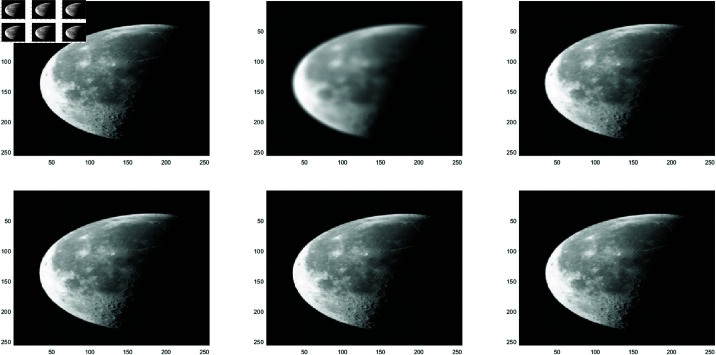
Moon image: (top left to right) original image, blurred image, and deblurred image using GMRES, (bottom left to right) deblurred image using *P*_*s*_*GMRES*, deblurred image using *P*_1_GMRES, and deblurred image using *P*_2_GMRES.

1. From Figs 2 and 3, it can be discerned that the eigenvalue spectra of $P_{1}^{-1}A$ and $P_{2}^{-1}A$ are notably more advantageous compared to that of $P_{S}^{-1}A$. Specifically, the eigenvalues of $P_{1}^{-1}A$ and $P_{2}^{-1}A$ tend to cluster around 1. Additionally, upon closer inspection of Fig 3, it is apparent that the eigenvalues of $P_{2}^{-1}A$ exhibit a tighter clustering around 1 compared to those of $P_{1}^{-1}A$ and $P_{S}^{-1}A$.
2. The effect of applying preconditioning becomes evident upon analyzing Fig 4. It is evident that the PGMRES methods, utilizing preconditioners *P*_1_ and *P*_2_, require notably fewer iterations compared to the standard GMRES (without preconditioning) and *P*_*S*_ to reach the desired accuracy. In contrast, the GMRES method necessitates more than 25 iterations to achieve the desired level of accuracy, The *P*_1_GMRES method, on the other hand, reaches the required precision in just a few iterations for matrices of size 512×512. A comparable trend is also evident for other matrix sizes.
3. Table 1 , 2 , 3 and 4 highlights that the preconditioners *P*_1_GMRES and *P*_2_GMRES not only achieve almost same PSNR values but also significantly reduces the number of iterations required. By leveraging the PGMRES algorithm with preconditioners *P*_1_ and *P*_2_, there is a notable reduction of over 30% in CPU time. As a result, the PGMRES method with *P*_1_ and *P*_2_ demonstrates superior overall performance in comparison to other technique.
4. Figs 5, 6, 7 and 8 provides a clear depiction of the enhanced quality achieved by PGMRES when using the preconditioners *P*_1_ and *P*_2_, showing a slight improvement in overall results.

**Example 2**

**Fig 9:**
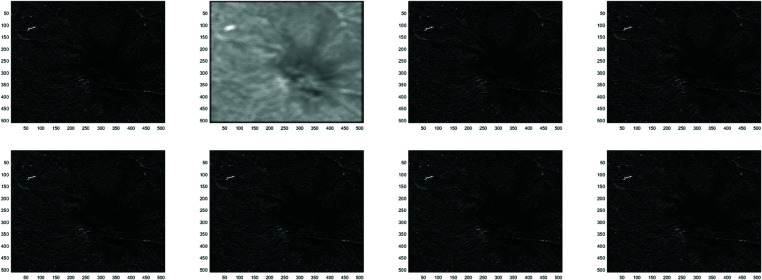
Yulin image: (top left to right) exact image, blurry image, deblurred image using *F*_1_ GMRES and deblurred image using *F*_2_ GMRES, (bottom left to right) deblurred image using GMRES, deblurred image using *P*_*s*_*GMRES*, deblurred image using *P*_1_GMRES, and deblurred image using *P*_2_GMRES.

**Fig 10:**
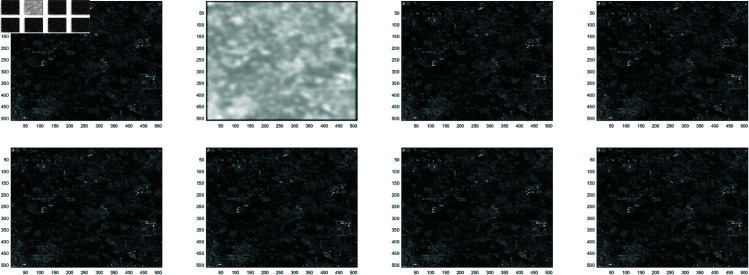
Beijing image: (top left to right) exact image, blurry image, deblurred image using *F*_1_ GMRES and deblurred image using *F*_2_ GMRES, (bottom left to right) deblurred image using GMRES, deblurred image using *P*_*s*_*GMRES*, deblurred image using *P*_1_GMRES, and deblurred image using *P*_2_GMRES.

**Fig 11:**
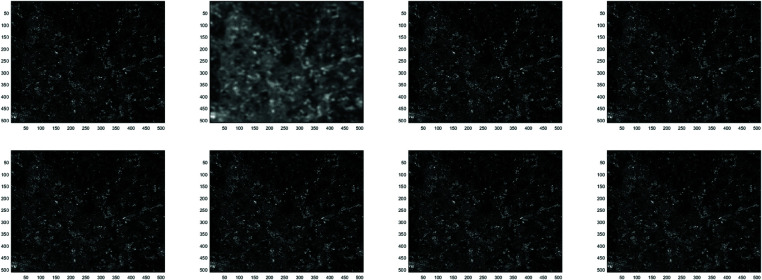
Milan image: (top left to right) exact image, blurry image, deblurred image using *F*_1_ GMRES and deblurred image using *F*_2_ GMRES, (bottom left to right) deblurred image using GMRES, deblurred image using *P*_*s*_*GMRES*, deblurred image using *P*_1_GMRES, and deblurred image using *P*_2_GMRES.

In this experiment, we utilized three hyperspectral images Yulin, Beijing, and Milan from the dataset used by Pan *et al*. [37] for image denoising. The blurring process was performed using the *ke*_−_*gen*(*N*,300,10) kernel. We also compared the results with the fractional-order TV-based algorithm (TFOV), specifically the F1GMRES and F2GMRES methods, as presented by Adel Al-Mahdi [7]. Figs 9, 10, and 11 illustrate the restored images, each with dimensions of 512×512. The blurry PSNR values for the Yulin, Beijing, and Milan images are 24.2248, 24.5601, and 23.07321, respectively. A halting criterion with a tolerance of $\text{tol}=1\;\times \;10^{-8}$ was applied in the numerical procedure. Further details on the experiment are provided in Table 5 .

**Table 5:** The numerical results for hyperspectral images.

Image		$F_{1}$ GMRES	$F_{2}$ GMRES	GMRES	$P_{S}$ GMRES	$P_{1}$ GMRES	$P_{2}$ GMRES
Yulin	PSNR	42.7756	42.7025	45.3978	45.0957	45.3399	45.3917
	CPU-Time	90.2582	91.2057	173.2673	83.6245	79.0256	76.3242
Beijing	PSNR	46.9351	46.3578	49.5635	49.6878	49.7511	49.7520
	CPU-Time	82.3567	80.6779	152.9636	68.3954	51.9190	52.8354
Milan	PSNR	44.7400	44.7921	47.1038	47.1524	47.1568	47.6391
	CPU-Time	83.6841	83.6705	161.2229	70.7832	56.6491	55.9395

**Remarks**

1. In this experiment, the MC-based method achieved higher PSNR compared to the TFOV-based methods. This can be observed from Table 5. Additionally, our proposed *P*_1_GMRES, and *P*_2_GMRES methods exhibited significantly faster CPU processing times while maintaining comparable PSNR values to other approaches. This demonstrates that our techniques are both more efficient and computationally faster for hyperspectral image deblurring.
2. Figs 9, 10, and 11 provides a clear depiction of the enhanced quality achieved by PGMRES when using the preconditioners *P*_1_ and *P*_2_, showing a slight improvement in overall results.

**Example 3**

**Fig 12:**
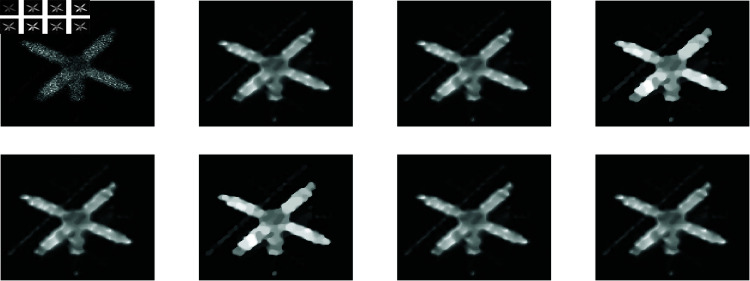
Satel image: (top left to right) blurry image, deblurred image using NFOV, deblurred image using *F*_1_ GMRES and deblurred image using *F*_2_ GMRES (bottom left to right) deblurred image using GMRES, deblurred image using *P*_*s*_*GMRES*, deblurred image using *P*_1_GMRES, and deblurred image using *P*_2_GMRES.

We used a satellite image from Chowdhury *et al*. [18] for this example, and we introduced observable artefact by applying blurring and Poisson noise to it. A Gaussian kernel defined by $\text{fspecial}{(}^{\prime }Gaussian^{\prime },9,\sqrt{3})$ was used to perform the blurring. We compared the outcomes with the TFOV-based methods (F1GMRES and F2GMRES) published by Adel Al-Mahdi [7] and the non-blind fractional order TV-based algorithm (NFOV) published by Chowdhury *et al*.[18]. Fig 12 displays the restored images, each with a size of 128×128. Two differnt sizes were used to test the methods: 128 and 64. This resulted in blurry PSNR values of 20.4559 and 20.2985, respectively.A halting criterion with a tolerance of $\text{tol}=1\;\times \;10^{-7}$ was employed in the numerical procedure. Table 6 has more details on the experiment.

**Table 6:** The numerical results for Satel image.

$n_{x}$	NFOV	$F_{1}$ GMRES	$F_{2}$ GMRES	GMRES	$P_{S}$ GMRES	$P_{1}$ GMRES	$P_{2}$ GMRES
64	26.9889	27.7972	27.8394	27.5737	27.8786	27.8453	27.8512
	35.3890	11.9244	11.0657	35.1226	9.9578	7.3935	6.9979
128	26.1827	26.4432	26.6563	26.1816	26.7542	26.9539	26.8822
	68.9287	21.7643	22.9515	69.5893	19.3978	16.9781	15.2640


**Remarks**

In this experiment, *P*_1_GMRES, and *P*_2_GMRES demonstrate significantly faster CPU processing times while achieving the same PSNR values as other methods. This indicates that our techniques are both more efficient and faster compared to GMRES, F1GMRES, F2GMRES, and NFOV methods.

## 6 Conclusions

This research presents a novel block preconditioning strategy specifically designed to address the 5×5 block matrix system arising from the discretization of the Euler-Lagrange equations in the context of curvature-driven image deblurring. The proposed preconditioners, *P*_1_ and *P*_2_, are assessed for performance and contrasted with the GMRES method and with some existing image deblurring approaches [3, 7, 18] . Theoretical insights indicate that the iterative approach achieves guaranteed convergence when paired with a suitable matrix decomposition method, underscoring the robustness and dependability of the proposed solution approach.

Besides verifying convergence, we perform a spectral analysis of the preconditioned matrices, offering a deeper understanding of their eigenvalues and spectral properties, which further reinforces the effectiveness of the preconditioning approach. The performance of the preconditioners is further demonstrated through a comprehensive numerical example, where the experimental findings consistently demonstrate improved performance. The approach introduced in this study enhances the quality of restored images, as evidenced by the improved PSNR values. Specifically, the proposed preconditioners (*P*_1_ and *P*_2_) demonstrate a modest improvement in convergence speed compared to the standard GMRES method, requiring fewer iterations and reduced computational time for rapid convergence and effective image deblurring. Moreover, the eigenvalues of the preconditioned matrices cluster tightly around 1, highlighting the robustness and efficiency of the proposed methodology.
